# Diffuse Uterine and Extrauterine Leiomyomatosis Presenting as a Large Abdominopelvic Mass Mimicking Malignancy

**DOI:** 10.7759/cureus.118917

**Published:** 2026-10-31

**Authors:** Zoiya Fatima, Jancy Ali, Nigar Mehtiyeva, Asma Fahad, Zuhdi Nagshabandi

**Affiliations:** 1 Obstetrics and Gynaecology, Latifa Women and Children Hospital, Dubai Health/Mohammed Bin Rashid University of Medicine and Health Sciences, Dubai, ARE; 2 Obstetrics and Gynaecology, Latifa Women and Children Hospital, Dubai Health, Dubai, ARE; 3 Obstetrics and Gynaecology, Dubai Health, Dubai, ARE

**Keywords:** abdominopelvic mass, abnormal uterine bleeding, case report, diffuse uterine leiomyomatosis, extrauterine leiomyomatosis

## Abstract

Diffuse uterine and extrauterine leiomyomatosis is an uncommon benign condition that can mimic malignancy on imaging. We report a 48-year-old multiparous woman presenting with abdominal pain and abnormal uterine bleeding. Imaging revealed a large heterogeneous uterine mass with peritoneal involvement. Total abdominal hysterectomy with bilateral salpingo-oophorectomy was performed. Histopathology confirmed diffuse leiomyomatosis. This rare entity poses diagnostic challenges and may lead to unnecessary aggressive treatment if misdiagnosed. Histopathology and immunohistochemistry are essential for accurate diagnosis. Awareness of this condition is crucial for appropriate management and avoidance of overtreatment.

## Introduction

Diffuse uterine leiomyomatosis (DUL) is a rare, benign condition characterized by the proliferation of numerous poorly defined, confluent smooth muscle nodules that replace most of the uterine parenchyma [1]. It differentiates itself from typical uterine leiomyomata through the involvement of the entire myometrium by numerous, ill-defined, often small and confluent, histologically benign smooth muscle nodules [2]. A number of symptoms, including abdominal discomfort, abnormal uterine bleeding, menorrhagia, dysmenorrhea, pelvic pressure, and infertility, are among the initial symptoms that are presented [3]. Management depends on the patient’s age and fertility wishes. Numerous case reports show uterine-sparing surgery in women requesting to preserve their fertility, with hysterectomy as the preferred option in women of advanced age [4,5].

Although DUL is a benign condition, it can be clinically and radiologically difficult to distinguish from malignant uterine neoplasms, making preoperative diagnosis a challenge [5,6]. Magnetic resonance imaging (MRI) is useful for preoperative evaluation; however, the definitive diagnosis is based on histopathological examination [1,6]. Due to the low incidence of this condition, awareness among clinicians is crucial in order to allow appropriate diagnosis and individualized management [4,6,7].

In this report, we present a rare case of DUL with associated extrauterine peritoneal leiomyomatosis, emphasizing the diagnostic challenges and management of this uncommon condition.

## Case presentation

A 48-year-old woman came to the emergency department with a complaint of generalized abdominal pain. She was para 3, with a history of three normal vaginal births. Upon further history, she also revealed abnormal uterine bleeding that had been persisting for almost a year. The clinical examination showed an abdominal mass, leading to additional assessment with contrast-enhanced computed tomography of the abdomen and pelvis. Imaging showed a large heterogeneous mass originating from the uterine myometrium, accompanied by a large peritoneal lesion. Furthermore, the spleen exhibited numerous indeterminate rounded hypodense lesions with mild enhancement (Figure 1).

**Figure 1:**
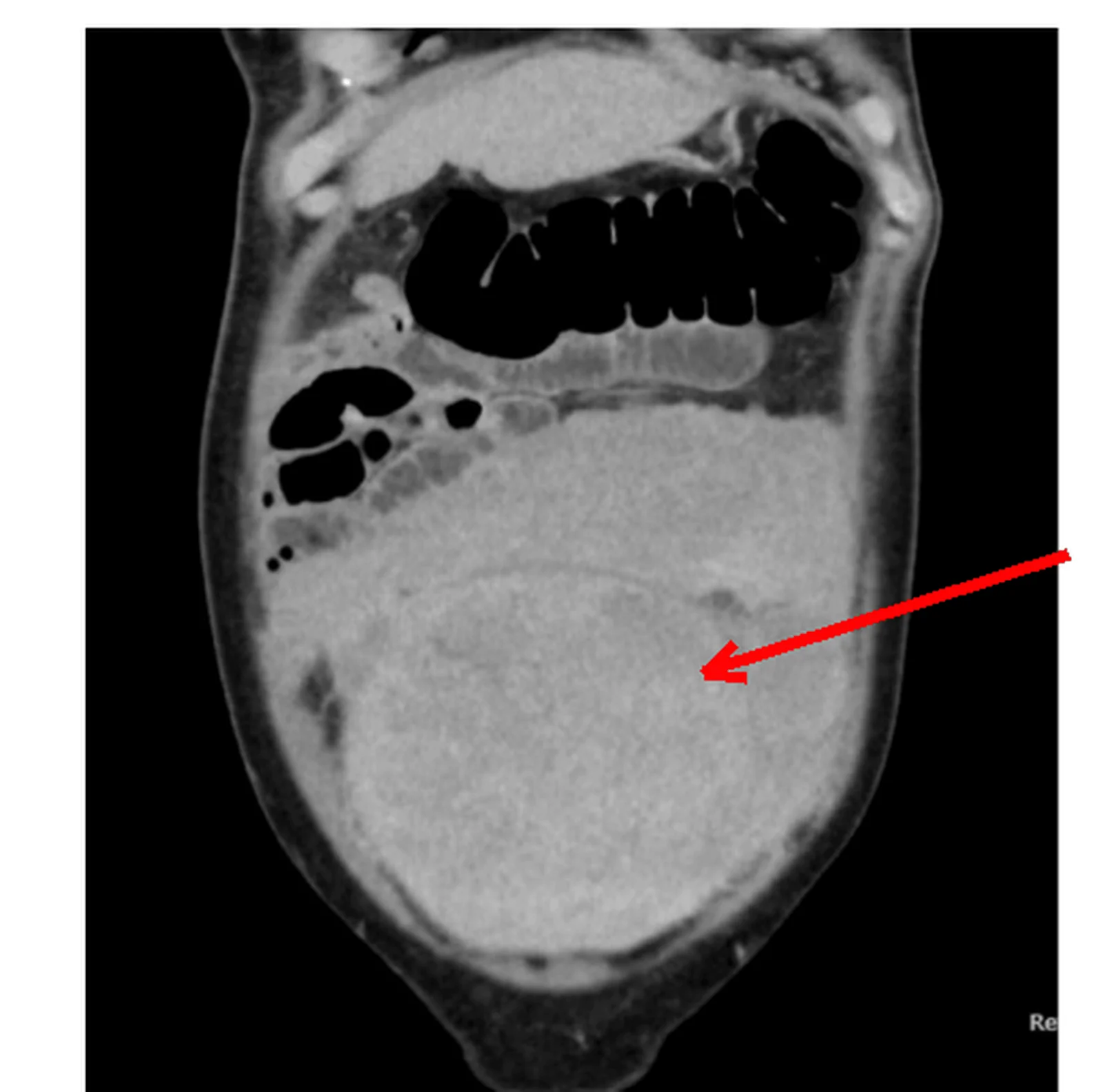
Large heterogeneous uterine myometrial mass accompanied by a large peritoneal lesion

A transabdominal pelvic ultrasound was performed to further analyze the pelvic findings, which revealed a large, heterogeneous, highly vascular mass arising from the posterior uterine myometrium, occupying a significant portion of both the abdomen and pelvis, measuring approximately 21 × 13 × 19 cm. No free intraperitoneal fluid was identified. Both ovaries were not visualized.

In view of the imaging results and the patient’s history and age, she was counseled and taken for total abdominal hysterectomy with bilateral salpingo-oophorectomy. Intraoperative findings revealed an enlarged uterus measuring 20-22 cm, accompanied by an abnormal lobulated mass approximately 10 x 12 cm in size. The mass appeared cystic, with a coarsely nodular and highly vascular nature, seemingly originating from the left ovary. An enlarged left infundibulopelvic ligament was noted, with the mass attached to the posterior uterine wall. Additionally, the mass exhibited mild adhesions to the small bowel, sigmoid colon, right lateral abdominal wall, and small bowel mesentery. Small deposits measuring approximately 1 x 1 cm were observed on the small bowel mesentery. The right ovary and tube appeared normal, as did the omentum and the upper abdomen (Figure 2 and Figure 3). Optimal debulking was achieved with no residual disease.

**Figure 2:**
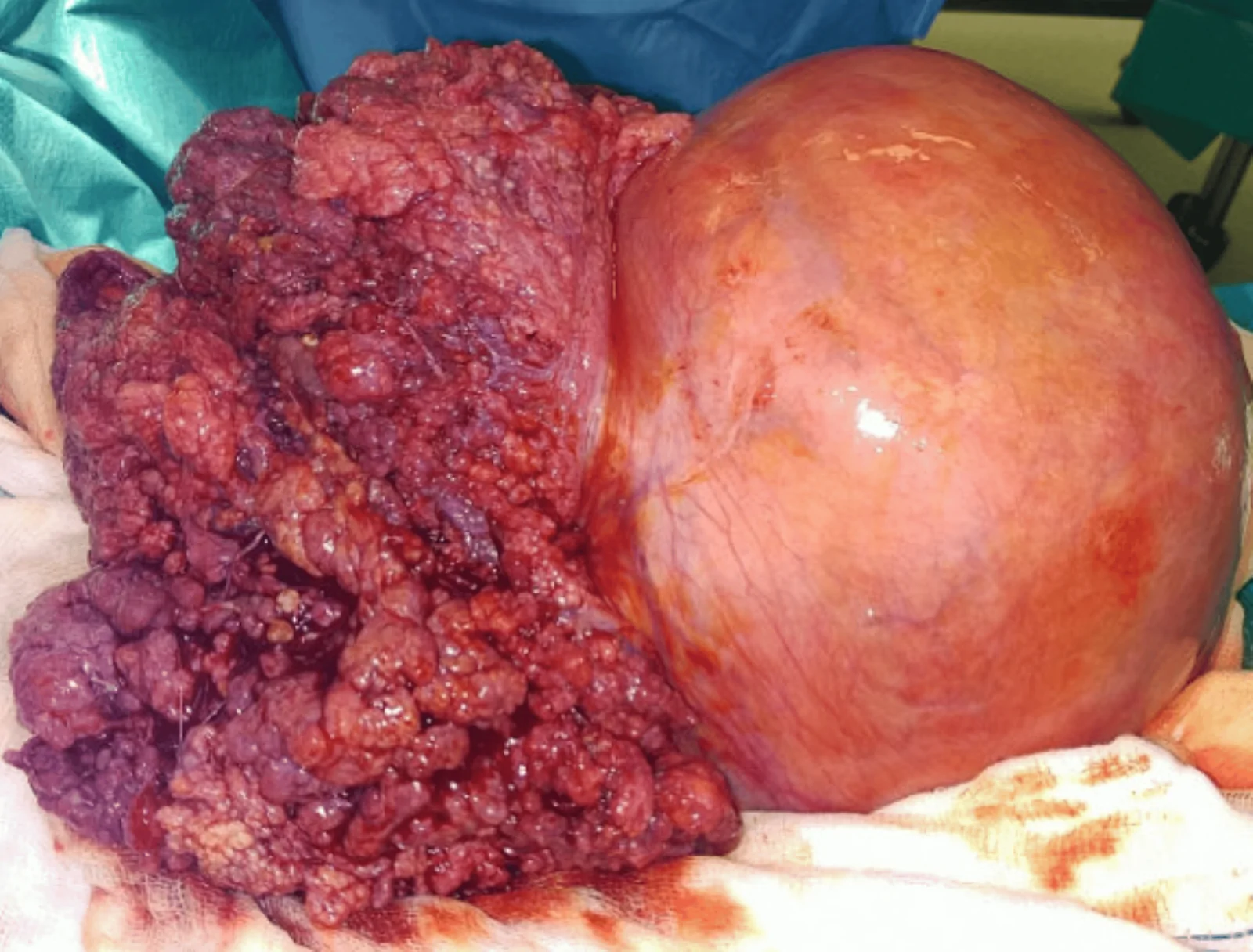
Enlarged uterus measuring 20–22 cm, accompanied by an abnormal lobulated mass approximately 10 x 12 cm in size. The mass appeared cystic, with a coarsely nodular and highly vascular nature

**Figure 3:**
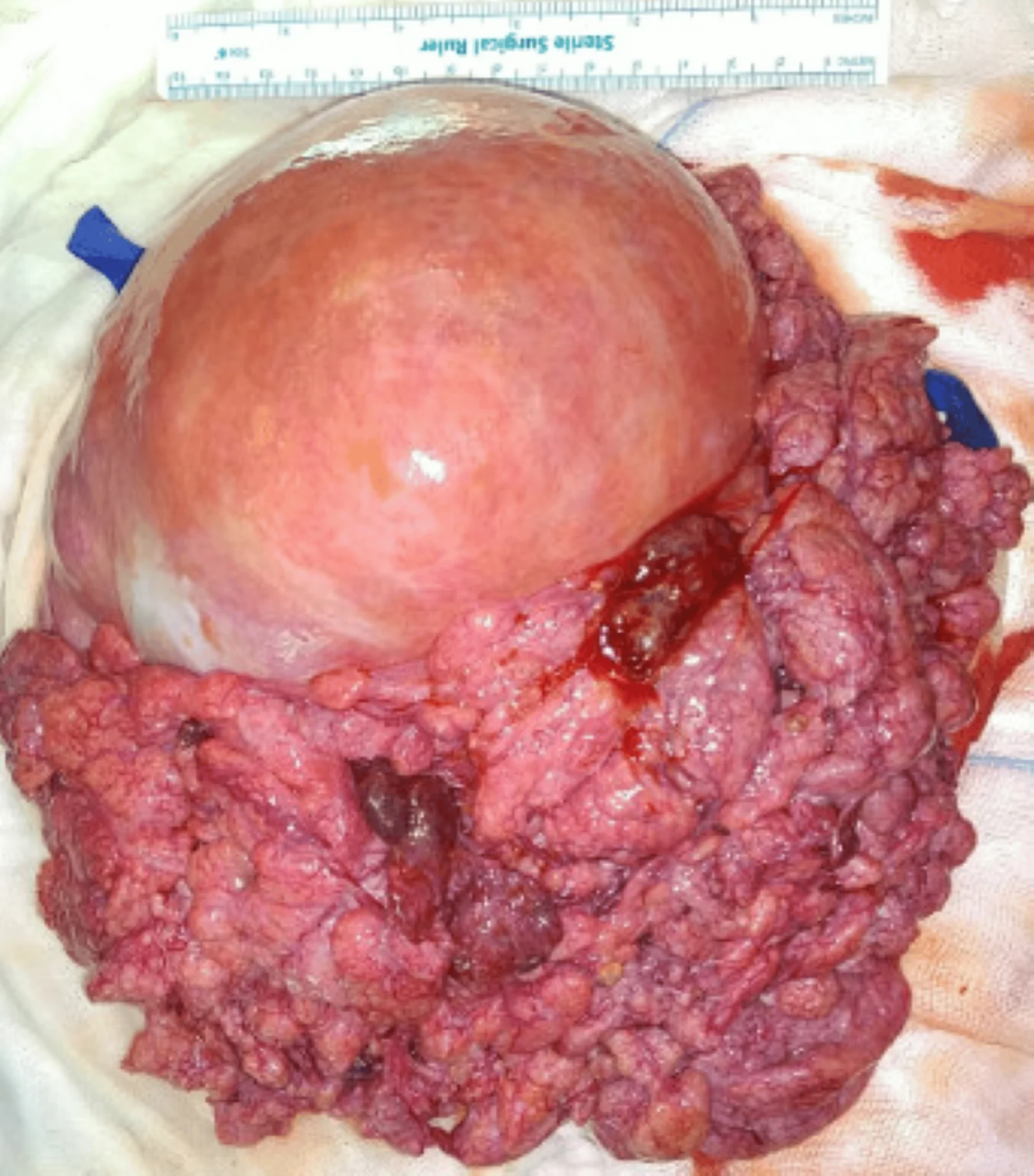
Uterus with leiomyomatosis mass

Histopathological examination revealed leiomyomatosis with proliferative nodules involving the peritoneum, consistent with features of extrauterine peritoneal leiomyomatosis. Additionally, the myometrium and serosa exhibited diffuse leiomyomatosis.

Microscopic description

The uterine myometrium (Figure 4) and peritoneum (Figure 5) are replaced by numerous poorly defined nodules of varying sizes. Some nodules appear circumscribed with rounded borders and plexiform morphology. These nodules are composed of smooth muscles highlighted by positive immunostaining for Desmin and smooth muscle actin. Individual cells are spindle-shaped with round, uniform, cigar-shaped nuclei. No mitosis, necrosis, or atypia are seen. Estrogen receptor shows nuclear staining in these nodules (Figure 6).

**Figure 4:**
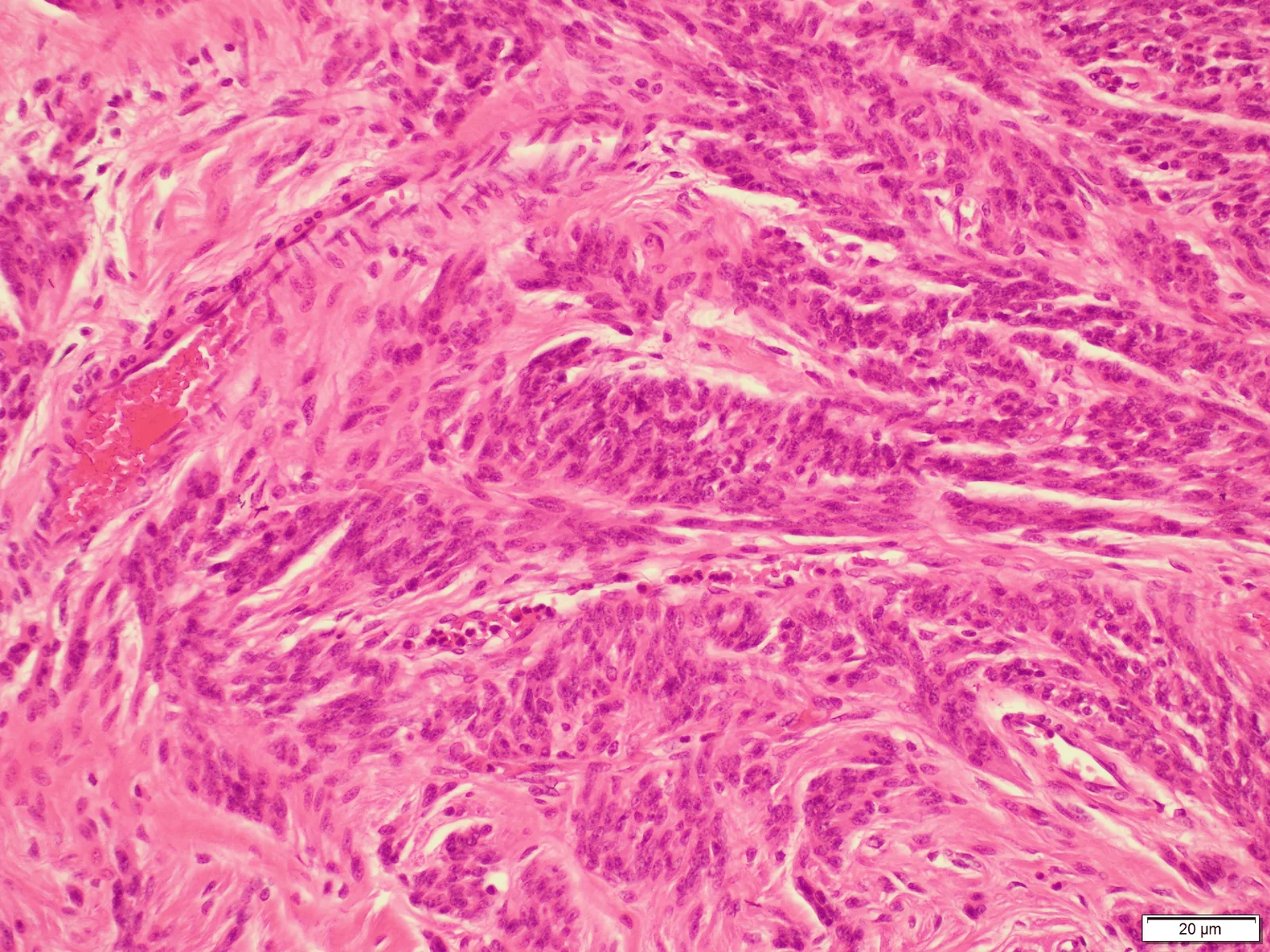
High-power view (20x) of the tumor in the uterus

**Figure 5:**
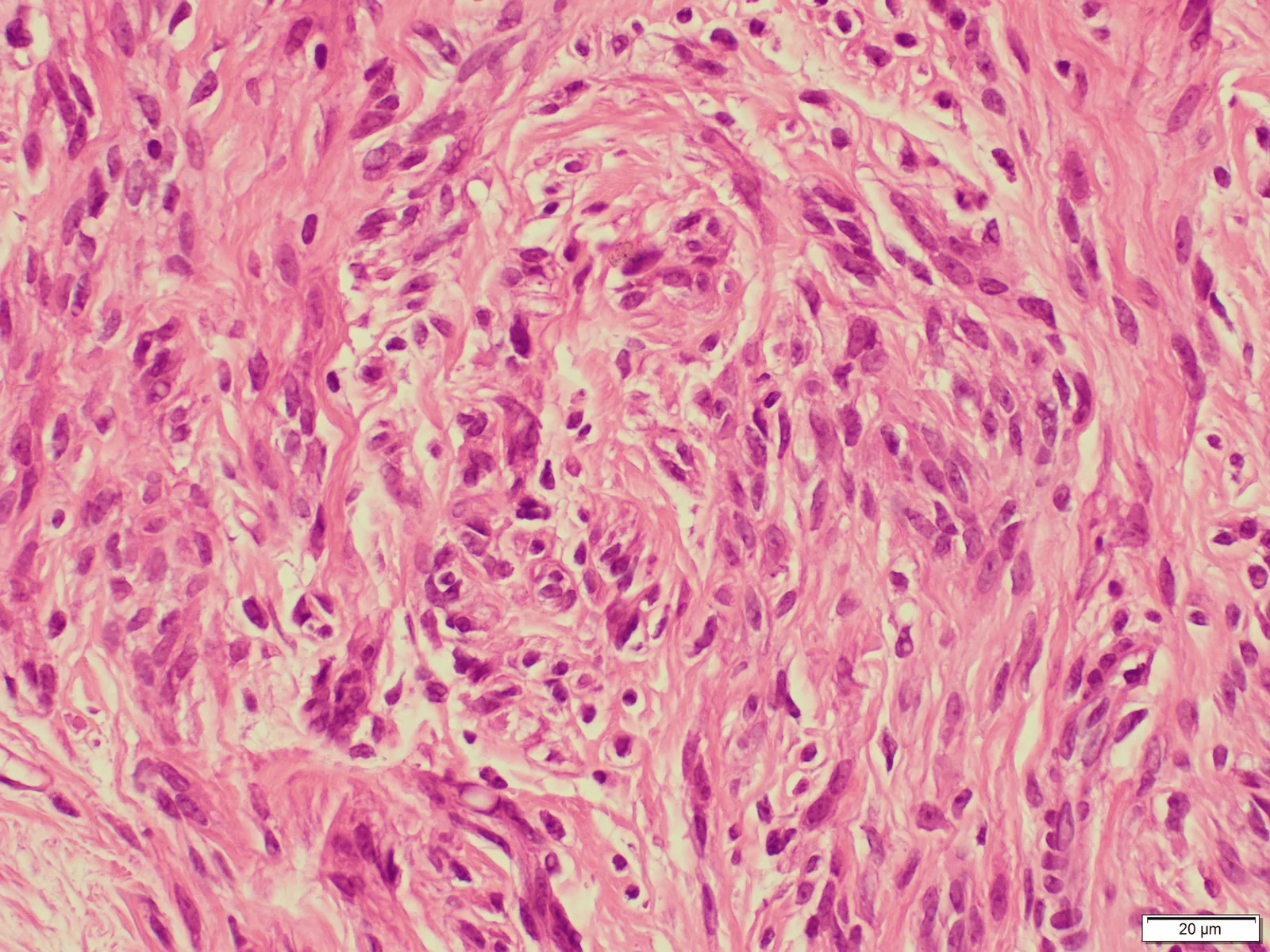
High-power 40x view of peritoneal nodules

**Figure 6:**
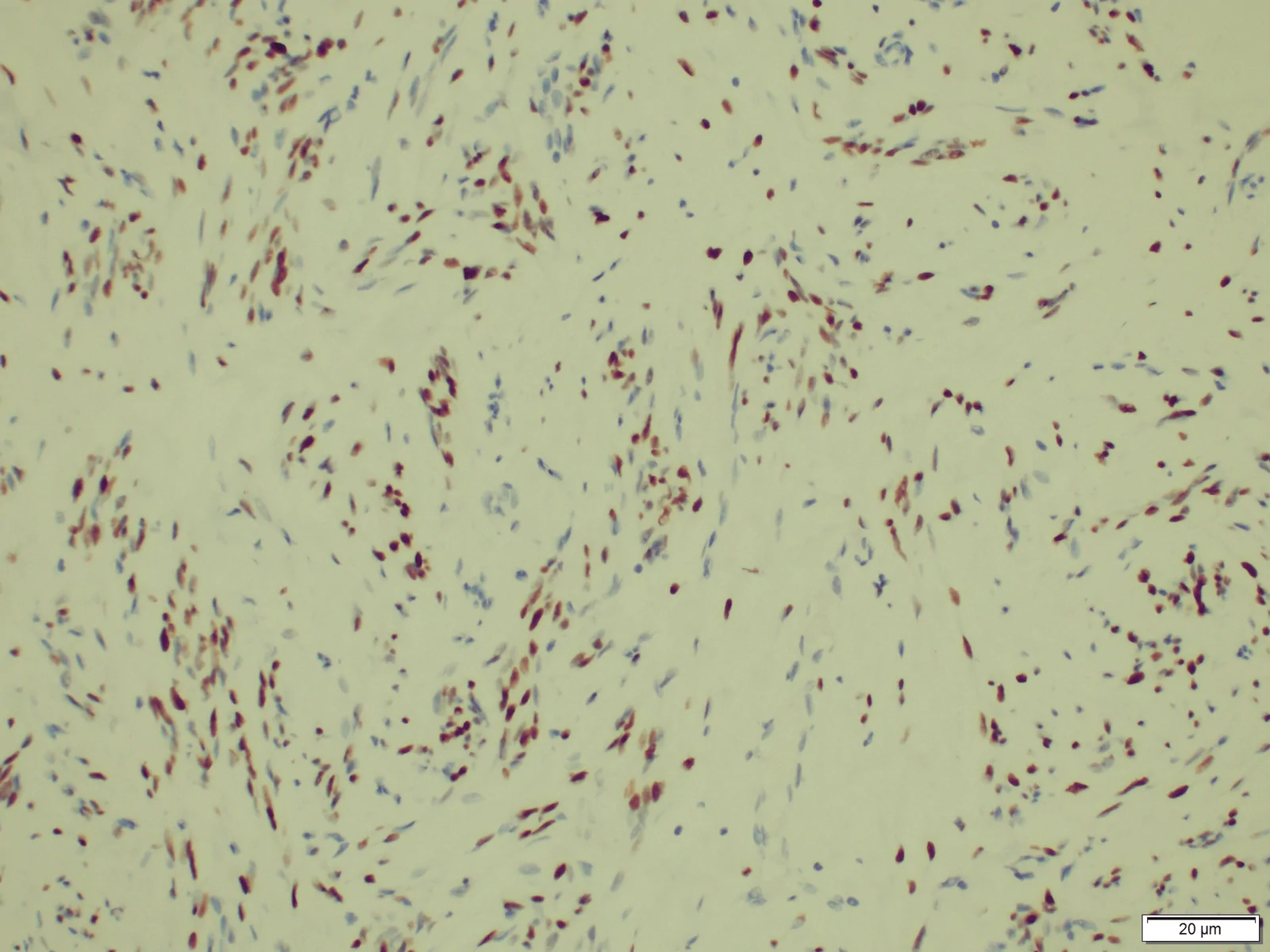
Medium-power 20x view of IHC estrogen receptors IHC: Immunohistochemistry

There was a discussion of the case in the tumor board and, considering the benign nature of the disease, she was scheduled for routine follow-up care after surgery. Postoperatively, the patient recovered well and was seen six weeks after surgery in a healthy condition.

## Discussion

In the literature, fewer than 50 sporadic cases of diffuse leiomyomatosis have been reported so far [5]. The initial presenting symptoms are abdominal pain, abnormal uterine bleeding, menorrhagia, dysmenorrhea, pelvic pressure, and infertility [5]. Our patient had similar symptoms of abdominal pain and abnormal uterine bleeding.

Preoperative misdiagnosis of DUL is common due to its rarity, and diagnosis is largely determined by preoperative MRI and postoperative pathology, resulting in inappropriate initial treatment of patients [6]. In the current era, total hysterectomy remains the most effective treatment for DUL. However, since it is common among young women of reproductive age, gynecologists are investigating ways to preserve reproductive function for those seeking fertility [7].

Chen et al. analyzed the clinical data for five DUL patients requesting fertility preservation, one of whom received high-intensity focused ultrasound (HIFU); one underwent laparoscopic myomectomy combined with hysteroscopic myomectomy; one underwent HIFU combined with HM and LRM; one underwent drug therapy combined with staged HM; and one underwent HIFU combined with staged HM and drug therapy, and treatment outcomes and pregnancy outcomes were examined. All five patients achieved successful pregnancy [8].

As the patient had completed her desired childbearing, a total abdominal hysterectomy was performed. Management decisions were individualized, taking into account the patient’s clinical profile, reproductive status, and overall treatment requirements.

## Conclusions

The diagnosis of uterine leiomyomatosis is difficult because it is a rare and benign condition. The treatment can be tailored to meet the individual needs and requirements of the patient, and a definitive diagnosis can be made through histopathology.

Due to its rarity, standardization of the treatment regimen and the establishment of an international registry of these rare cases would be helpful in the future. This case report is limited by its single-patient design and lack of long-term follow-up. Findings may not be generalizable to all patients with diffuse leiomyomatosis.
