# Correction: Diffuse Uterine and Extrauterine Leiomyomatosis Presenting as a Large Abdominopelvic Mass Mimicking Malignancy

**DOI:** 10.7759/cureus.c501

**Published:** 2026-11-09

**Authors:** Zoiya Fatima, Jancy Ali, Nigar Mehtiyeva, Asma Fahad, Zuhdi Nagshabandi

**Affiliations:** 1 Obstetrics and Gynaecology, Latifa Women and Children Hospital, Dubai Health/Mohammed Bin Rashid University of Medicine and Health Sciences, Dubai, ARE; 2 Obstetrics and Gynaecology, Latifa Women and Children Hospital, Dubai Health, Dubai, ARE; 3 Obstetrics and Gynaecology, Dubai Health, Dubai, ARE

This article has been corrected to change the description of the hysterectomy from “total laparoscopic hysterectomy” to “total abdominal hysterectomy”.

